# 

**Published:** 1887-03

**Authors:** 


### International Medical Congress.—Trans-Atlantic Rates.

In an official notice from the Chairman of the Committee of Arrangements at Washington, he says that reliable arrangements have been made by which members wishing to attend the International Medical Congress in Washington September 5, 1887, can be accommodated by the following steamship lines at the liberally reduced rates mentioned, viz:

Red Star Line.—$100, Antwerp—New York and return.

Inman Line.—$100, Liverpool—New York and return.

Hamburg Line.—$90, Hamburg—New York and return.

Royal Netherland.—$80, Amsterdam—New York and return.

Since that notice we have received authentic information that the several lines named have consented to extend the same rates to the families of members, as the following letter shows:

WASHINGTON, D C., FEB. 14, 1887.

A. Y. P. GARNETT, M. D., *Chairman of the Committee of Arrangements of the International Medical Congress:*

*Dear Doctor*:—I am happy to inform you that, through the instrumentality of Mr. Edward F. Droop, agent in this city for the Lines, who has manifested so much interest in this matter, we have been able to secure from the Hamburg-American, the Red Star, and the Inman Lines the offer of the same reductions for the families of members of the Congress as those I have already reported for the members themselves. Very Truly Yours,

J. W. H. LOVEJOY,

Chairman Com. on Transportation.

To aid the work of the Committee on Transportation, the State Department of our Government at Washington has kindly instructed the resident U. S. Consuls at European ports from which the steamships leave, to actively aid in ascertaining the number of those wishing to avail themselves of the reduced rates offered.

### CONTENTS.

COMMUNICATIONS.

### THE DENTAL REGISTER,

**A MONTHLY MAGAZINE DEVOTED TO THE INTERESTS OF THE**

### DENTAL PROFESSION.

*Terms Reduced to $2.00 Per Year. Single Copies, 25 Cents.*

**EDITED BY PROF. J. TAFT, D.D.S.**

—AND—

**Published by SAM'L A. CROCKER & CO., Ohio Dental and Surgical Depot.**

The volume commences in January and closes in December.

The REGISTER being issued monthly is always fresh, therefore Indispensable to the practitioner who desires to keep posted up with the new inventions and improvements which are daily developed. Neither expense nor effort will be spared to give value and variety to its contents. Articles will be illustrated with well executed engravings whenever they will add to the interest or assist to explanation.

Its extensive circulation and frequency of issue make it one of the BEST DENTAL ADVERTISING MEDIUMS in the United States.

All subscriptions must begin with January or July.

Local or special notices, five lines or less, will be inserted for $3 each insertion; over five lines, 50 cts. per line.

All advertising bills payable in advance, or at furthest, after the issue of the first number containing the advertisement. All transient advertisements to be paid for in advance.

☞CONTRIBUTIONS SOLICITED.

Exclusively Editorial Communications should be addressed to the Editor.

The latest number of the REGISTER may be ordered with or without other goods at any time, and all letters should be addressed to

**SAM'L A. CROCKER & CO., Ohio Dental and Surgical Depot, Cincinnati, O.**

